# Illuminating calcium and potassium dynamics with red fluorescent sensors

**DOI:** 10.1371/journal.pbio.3003460

**Published:** 2025-10-21

**Authors:** Lan Geng, Yulong Li

**Affiliations:** 1 State Key Laboratory of Membrane Biology, Peking University School of Life Sciences, Beijing, China; 2 PKU-THU Center for Life Sciences, New Cornerstone Science Laboratory, Beijing, China; 3 PKU-IDG/McGovern Institute for Brain Research, Beijing, China

## Abstract

Despite recent advances in green genetically encoded ion indicators, developments in red-shifted indicators have been slower. This Primer explores two recent studies in PLOS Biology that engineer red fluorescent indicators for visualizing potassium and calcium ion dynamics in culture and in vivo.

## Teaser

A pair of recent studies in *PLOS Biology* introduces red fluorescent genetically encoded indicators for calcium and potassium, extending the optical toolkit for tracking these essential signals in the living brain.

**Fig 1:**
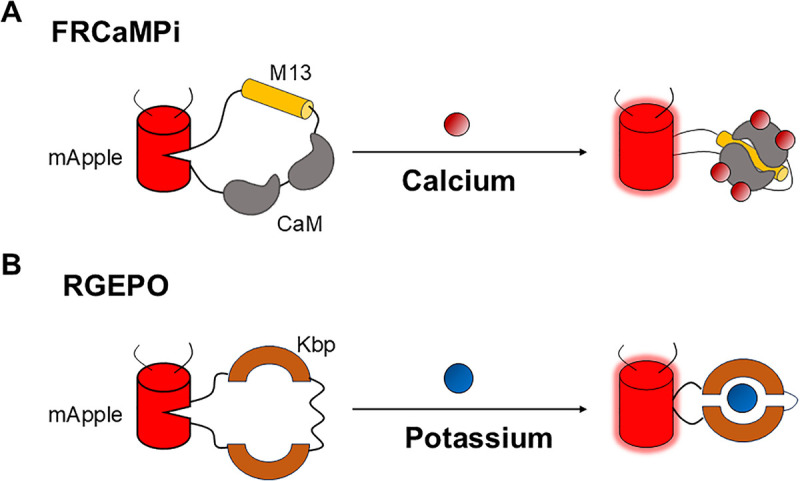
Principles of FRCaMPi and GEPOs. **(A)** Principle of FRCaMPi. The calcium-sensing domain (CaM and M13) is inserted within mApple. After calcium binding, the brightness of the sensor will increase. CaM: calmodulin. **(B)** Principle of RGEPO. The potassium-sensing domain is inserted within mApple. After potassium binding, the brightness of the sensor will increase. Kbp, potassium binding protein.

Calcium and potassium ions play essential roles in numerous physiological processes, including neuronal signaling, muscle contraction, and cellular homeostasis. Detecting these ions under physiological conditions is crucial for understanding their functions in both health and disease. Among the available detection tools, genetically encoded indicators stand out for their ability to monitor ion dynamics in real time with minimal invasiveness and high spatial and temporal resolution. Notably, single fluorescent protein-based sensors offer several advantages over sensors based on Förster resonance energy transfer, including a higher signal-to-noise ratio (SNR) and compatibility with multicolor imaging. These sensors are typically composed of two modules: an ion-binding domain that responds to changes in ion concentration, and a fluorescent protein that transduces this biochemical signal into an optical readout. After ion binding, the fluorescence intensity of the sensor will increase (Fig 1).

While GFP-based calcium and potassium indicators have been common [1–3], red-shifted sensors are particularly attractive. Their longer wavelengths lead to reduced phototoxicity, better penetration for deep tissue imaging, and the ability for multiplex imaging with indicators in other channels. Although several red calcium indicators have been reported [4–6], their sensitivity remains suboptimal. To address these limitations, Zhou and colleagues developed two improved red calcium sensors: FRCaMPi and its soma-targeted variant SomaFRCaMPi [7]. Red potassium sensors, by contrast, had not previously been available until now. To fill this gap, Yang and colleagues developed the first red genetically encoded fluorescent potassium indicators, RGEPO1 and RGEPO2 [8]. These two studies are recently published in *PLOS Biology*, which together represent a significant expansion of the genetically encoded red ion sensor toolkit.

Many existing calcium indicators are built by inserting the calcium-sensing domains M13 and calmodulin (CaM) between the N- and C-termini of a fluorescent protein [4–6,9]. In contrast, some of the best-performing designs adopt an inverted topology, in which the sensing domains are embedded within the fluorescent protein, resulting in improved affinity and sensitivity [10]. Zhou and colleagues applied this inverted design strategy to the previously developed FRCaMP [9], creating FRCaMPi (Fig 1A). The new sensor demonstrated approximately 2-fold higher calcium affinity compared to FRCaMP, while maintaining a similar dynamic range in cultured cells. Notably, FRCaMPi exhibited greater sensitivity and improved dynamic performance than other red calcium indicators in mammalian cell cultures, despite its slower off-kinetics. Having developed FRCaMPi, the authors want to further improve the sensors’ performance in living animals. Complementary to topological engineering, soma-targeting of genetically encoded calcium indicators (GECIs) is known to improve the effectiveness of population calcium imaging at single-neuron resolution in vivo due to reduced neuropil contamination, which is signal arising from surrounding axons and dendrites that can obscure soma-specific fluorescence, and enhanced sensor brightness in soma region. As a result, they engineered a soma-targeted variant by fusing the RPL10 peptide to the C-terminus of FRCaMPi, called SomaFRCaMPi. In cultured cells, SomaFRCaMPi maintained high sensitivity and outperformed other soma-targeted red calcium indicators.

The true strength of these sensors was demonstrated in living animals. In zebrafish larvae and mice, the authors showed that both FRCaMPi and SomaFRCaMPi displayed larger fluorescence changes in response to calcium activity compared to jRGECO1a, a widely used red calcium indicator [5]. Importantly, SomaFRCaMPi provided high SNR and improved single-cell resolution by reducing background fluorescence from surrounding processes. Under one-photon (1P) wide-field imaging in mice, SomaFRCaMPi enabled detection of neuronal activity in densely labeled populations. Under two-photon (2P) imaging, it reliably reported activity from deeper cortical layers. Moreover, SomaFRCaMPi helped minimize neuropil contamination in both 1P and 2P imaging, showcasing the advantages of soma-targeted calcium sensors. Notably, when evaluated side-by-side with RiboL1-jGCaMP8s in the brainstem of living animals, SomaFRCaMPi achieves signal amplitudes comparable to state-of-the-art soma-localized green indicators under identical conditions. This marks an important milestone for red GECIs, addressing the historical bottleneck of lower sensitivity relative to green sensors and highlighting the practical advantages of red fluorescence for imaging subcortical regions such as the brainstem.

Yang and colleagues developed the first red genetically encoded fluorescent potassium indicators, RGEPO1 and RGEPO2. The RGEPO sensors were engineered by swapping calcium-binding domains in FRCaMPi with Hv-Kbp, a potassium-binding protein domain. (Fig 1B). The RGEPO series was developed and optimized through a combination of directed molecular evolution in *Escherichia coli* and mammalian cells to improve sensor performance across biological systems.

Characterization in cultured cells revealed that the indicators exhibited robust responsiveness and high specificity toward potassium ions. Moreover, the sensors successfully reported potassium dynamics in both intracellular and extracellular compartments of cultured neurons and astrocytes. To further investigate the molecular basis of potassium sensing, the authors performed molecular dynamics simulations, which provided mechanistic insights into the ion-binding interactions within the RGEPO structure. These simulations revealed key conformational changes upon potassium binding, which support the mechanistic basis for the sensors’ observed selectivity and sensitivity.

Their utility was further confirmed in acute brain slices and in living mice. Notably, RGEPOs were used to simultaneously detect potassium and calcium dynamics by combining them with the green calcium indicator GCaMP6f, demonstrating excellent spectral compatibility with green fluorescent sensors. Notably, in a kainic acid-induced seizure model, RGEPO1 and RGEPO2 were able to report extracellular and intracellular spreading K⁺ waves, respectively, with distinct arrival times across individual cell, illustrating their utility in capturing spatially and temporally resolved potassium fluctuations across brain tissue. These findings confirm that RGEPOs can visualize both intracellular and extracellular potassium activity in real time—a significant advancement for potassium imaging. Although these sensors represent a major leap forward, further optimization of their dynamic range, response kinetics, and sensitivity will be essential to broaden their applications in complex physiological settings.

Taken together, these two studies report exciting progress in red-shifted ion imaging. By introducing improved calcium sensors (FRCaMPi and SomaFRCaMPi) and the first red potassium sensors (RGEPO1 and RGEPO2), they significantly expand the imaging toolkit for probing cellular activity in the brain and other tissues. These sensors are expected to enable deep-tissue, multiplexed, and minimally invasive imaging, opening new possibilities for studying neural dynamics and ion signaling across various biological systems. Importantly, these red indicators enable simultaneous imaging of calcium or potassium dynamics together with neurotransmitter or neuromodulator signals, which can be detected using green fluorescent sensors. Such combinations could allow researchers to track neurochemical events, such as small-molecule neurotransmitter or neuropeptide release, in parallel with neuronal activity. Monitoring these signals in vivo under physiological or pathological conditions would offer powerful insights into the mechanisms of neuromodulation across diverse brain regions. However, a key limitation remains: mApple-based sensors, like jRGECO1a, are susceptible to photoactivation by blue light [5]. This effect poses challenges when these sensors are used in combination with blue light-driven optogenetic tools. Therefore, future efforts to minimize photoactivation, while further enhancing photostability, ion selectivity, and dynamic range, will be critical for improving the utility and reliability of these sensors in diverse experimental conditions.

## Abbreviations

**:**
GECIs: genetically encoded calcium indicators
SNR: signal-to-noise ratio
